# The anomalous state of Uranus’s magnetosphere during the Voyager 2 flyby

**DOI:** 10.1038/s41550-024-02389-3

**Published:** 2024-11-11

**Authors:** Jamie M. Jasinski, Corey J. Cochrane, Xianzhe Jia, William R. Dunn, Elias Roussos, Tom A. Nordheim, Leonardo H. Regoli, Nick Achilleos, Norbert Krupp, Neil Murphy

**Affiliations:** 1 NASA Jet Propulsion Laboratory, California Institute of Technology, Pasadena, CA USA; 2 Dept. of Climate and Space Sciences and Engineering, University of Michigan, Ann Arbor, MI USA; 3 Dept. of Physics and Astronomy, UCL, London, UK; 4 Max Planck Institute for Solar System Research, Göttingen, Germany; 5 Applied Physics Laboratory, John Hopkins University, Laurel, MD USA

**Keywords:** Giant planets, Magnetospheric physics, Planetary science

## Abstract

The Voyager 2 flyby of Uranus in 1986 revealed an unusually oblique and off-centred magnetic field. This single in situ measurement has been the basis of our interpretation of Uranus’s magnetosphere as the canonical extreme magnetosphere of the solar system; with inexplicably intense electron radiation belts and a severely plasma-depleted magnetosphere. However, the role of external forcing by the solar wind has rarely been considered in explaining these observations. Here we revisit the Voyager 2 dataset to show that Voyager 2 observed Uranus’s magnetosphere in an anomalous, compressed state that we estimate to be present less than 5% of the time. If the spacecraft had arrived only a few days earlier, the upstream solar wind dynamic pressure would have been ~20 times lower, resulting in a dramatically different magnetospheric configuration. We postulate that such a compression of the magnetosphere could increase energetic electron fluxes within the radiation belts and empty the magnetosphere of its plasma temporarily. Therefore, the interpretation of Uranus’s magnetosphere as being extreme may simply be a product of a flyby that occurred under extreme upstream solar wind conditions.

## Abstract (web summary)

A reanalysis of the Voyager 2 flyby of Uranus shows that it occurred during an extreme compression of the planet’s magnetosphere by the upstream solar wind. This would have had significant effects on the measurements made during the flyby.

## Main

All previous magnetospheric analyses of the Voyager 2 flyby of Uranus that have utilized the upstream solar wind conditions have focused on the data acquired a few hours before the first bow shock crossing. Therefore, the picture that has been built of the planet’s magnetosphere is representative of the solar wind conditions that existed only during the flyby. This includes a solar wind number density (*n*) of 0.05 cm^−3^ and a velocity (*ν*) of 470 km s^−1^; with a resulting dynamic pressure *P*_dyn_ = *mnν*^2^, of 0.018 nPa (ref. ^1^) (assuming^2^ that the solar wind mass *m* is that of 97% protons and 3% He^++^). The vast majority of subsequent analyses and theoretical modelling of the Uranian magnetosphere has focused on these upstream conditions, including mission planning for a future flagship mission^3–7^.

**Fig. 1:**
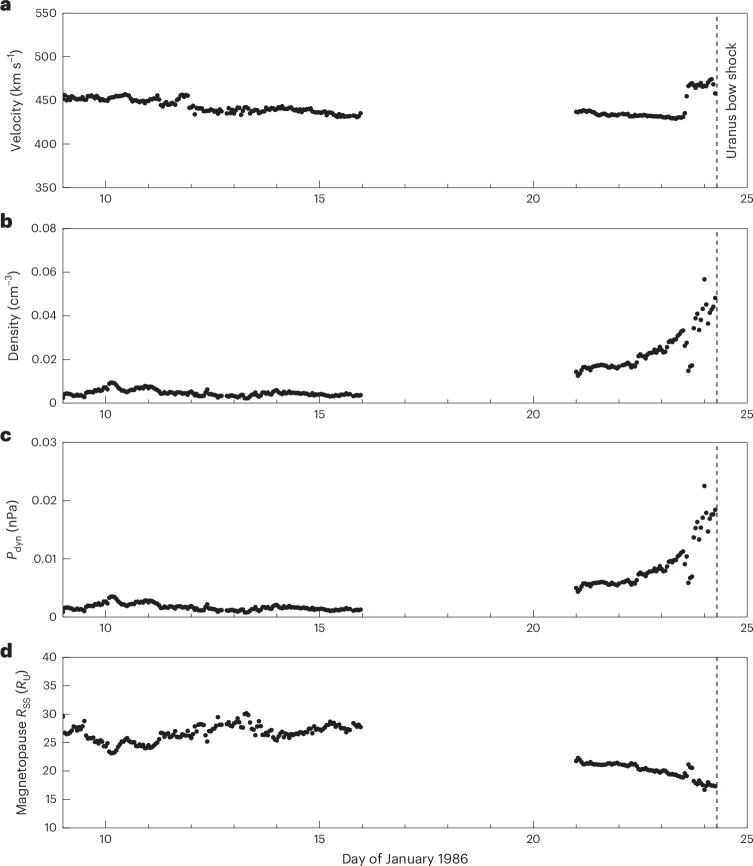
Voyager 2 in situ measurements of solar wind conditions upstream of Uranus, before the flyby. **a**–**d**, Measurements were made by the PLS. The dashed vertical line shows when Voyager 2 crossed Uranus’s bow show on 24 January 1986 at 07:28 ut. The solar wind velocity (**a**), solar wind density (**b**), solar wind dynamic pressure (**c**) and the estimated expected magnetopause subsolar standoff distance (*R*_SS_) at Uranus shown in planetary radii where *R*_U_ is equal to 25,559 km (**d**) are shown. A data-gap is present between day 16 and 21. Source data

Figure 1 shows a fortnight of the Voyager 2 solar wind data upstream of Uranus before the spacecraft’s first bow shock crossing on the 24th of January 1986. A data-gap is present between day 16 and 21. Figure 1a–c shows the solar wind velocity, density and dynamic pressure. The dashed line on day 24 shows the location of the Uranus bow shock crossed by Voyager 2. During the final few hours before the bow shock crossing (close to the dashed line), *P*_dyn_ is indeed at the quoted 0.018 nPa level. However, we can see that both the density and dynamic pressure were steadily increasing for 2 days (including a modest increase in *ν*) before the flyby, from ~0.005 nPa to 0.018 nPa (an almost fourfold increase from day 22), after having been steady at 0.005 nPa for a day (on day 21). It can also be seen that eight days before the flyby started, *P*_dyn_ was even lower at 0.001 nPa, with a minimum at 0.00078 nPa on day 13. Such low-pressure values are ~18 to ~23 times lower than during the Uranus flyby itself. Furthermore, when Voyager 2 had exited the Uranian magnetosphere (that is, the outbound bow shock), *P*_dyn_ was even higher than before the inbound crossing, at 0.028 nPa, which indicates that the major solar wind *P*_dyn_ enhancement continued and even increased throughout the Uranus flyby.

It is important to note that a minority of past discussions have commented on the upstream solar wind conditions at Uranus. For example, Gurnett et al.^8^ state that “the solar wind density was unusually high”. The radio and UV auroral emissions were shown to be increased by major enhancements in the solar wind conditions^9,10^, and analysis of the current sheet crossing suggests that this enhancement possibly ‘relaxed’ while Voyager 2 was inside Uranus’s magnetosphere^11^. These discussions, however, did not analyse the upstream solar wind conditions in detail, nor did they put them in context of the flyby conditions at Uranus and the possible subsequent effects on the discoveries made by Voyager 2.

## Magnetopause subsolar standoff location

In Fig. 1d, we have estimated how different *P*_dyn_ values would affect the subsolar standoff distance (*R*_SS_) of the magnetopause (see Methods for more details about this estimation), which provides an approximate measure of the size of Uranus’s global magnetosphere. Figure 1d shows that, on day 16, the magnetopause *R*_SS_ was estimated to be at a location of ~28 *R*_U_ from the planet, and on day 21 this was compressed to ~22 *R*_U_, before Voyager 2 finally observed a subsolar magnetopause at ~17 *R*_U_ on day 24 (where *R*_U_ is Uranus’s radius and equal to 25,559 km). This represents a substantial (~40%) change in the subsolar magnetopause location, and a substantial reduction in the volume of the dayside magnetosphere (~78%; assuming a simple hemispherical dayside magnetosphere).

**Fig. 2:**
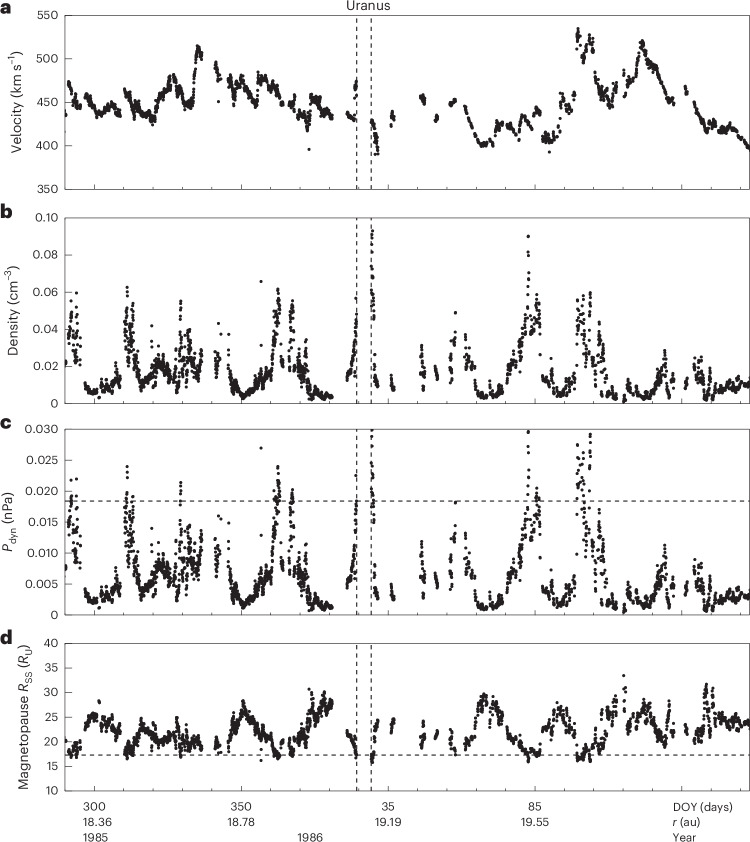
Voyager 2 in situ measurements of solar wind conditions at Uranus’s orbit. **a**–**d**, Voyager 2 crossed Uranus’s orbital path from day of year (DOY) 290 of 1985 until DOY 158 of 1986. This corresponds to Uranus’s perihelion and aphelion heliocentric radial distances (*r*) of 18.28au and 20.09 au, respectively. This figure is in the same format as Fig. 1. The solar wind velocity (**a**), solar wind density (**b**), solar wind dynamic pressure (**c**) and estimated expected magnetopause subsolar standoff distance (*R*_SS_) at Uranus (**d**) are shown. The Uranus flyby (from first to final bow shock crossing) is represented by the dashed vertical lines and data from that time period is not shown and not used for this analysis. The horizontal dashed lines show the *P*_dyn_ and *R*_SS_ that were observed by Voyager 2 moments before crossing Uranus’s inbound bow shock on 24 January 1986. Source data

Figure 2 shows the solar wind conditions and *R*_SS_ for the entire interval that Voyager 2 spent in the heliosphere at the range of radial distances at which Uranus orbits the Sun (in the same format as Fig. 1). Uranus’s orbit has an eccentricity of 0.047, with a perihelion located at 18.28 au and an aphelion at 20.09 au. Figure 2 shows when Voyager 2 was located within this range (for details regarding why no solar wind propagation is required for this dataset, please see Methods and Supplementary Information, including Supplementary Figs. 1 and 2).

Figure 2 illustrates that there was a wide range of upstream conditions at Uranus, which would have dramatically affected the magnetopause boundary and thus the global configuration of the magnetosphere. Based on these measurements, we estimate an average *P*_dyn_ of ~0.006 nPa. Upon exiting the magnetosphere, the expected subsolar magnetopause location was at ~16 *R*_U_, which shows that, while Voyager 2 was inside Uranus’s magnetosphere, it was compressed even further towards the planet. Observed maximum and minimum *P*_dyn_ values of 0.031 nPa and 0.00043 nPa correspond to expected magnetopause standoff distances of 15.8 *R*_U_ to 33.5 *R*_U_, respectively. It is also evident that the solar wind at Uranus varies on a timescale close to the Sun’s rotation period (~27 days). This suggests that the upstream conditions at Uranus (during the Voyager 2 era at solar minimum) may have typically consisted of regular passing corotating interaction regions (CIRs).

**Fig. 3:**
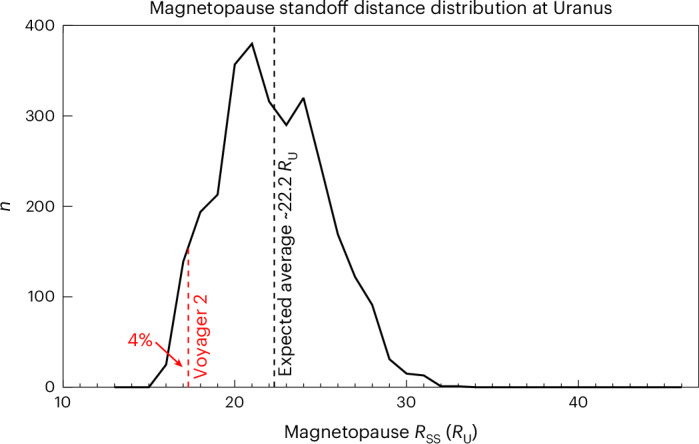
Magnetopause standoff distance at Uranus. The expected distribution of magnetopause subsolar standoff distances (*R*_SS_) estimated from upstream conditions at Uranus shown in Fig. 2d. The *y* axis shows the number ‘*n*’ of datapoints from Fig. 2d (that is, the number of occurrences) for a particular subsolar magnetopause location. The dashed red line shows the subsolar location observed by Voyager 2 of 17.3 *R*_U_. A magnetopause location of 17.3 *R*_U_ or lower is expected only 4% of the time. The mean expected magnetopause subsolar standoff location is 22.2 *R*_U_. The interquartile range is expected to be between 20 and 25 *R*_U_. Source data

**Fig. 4:**
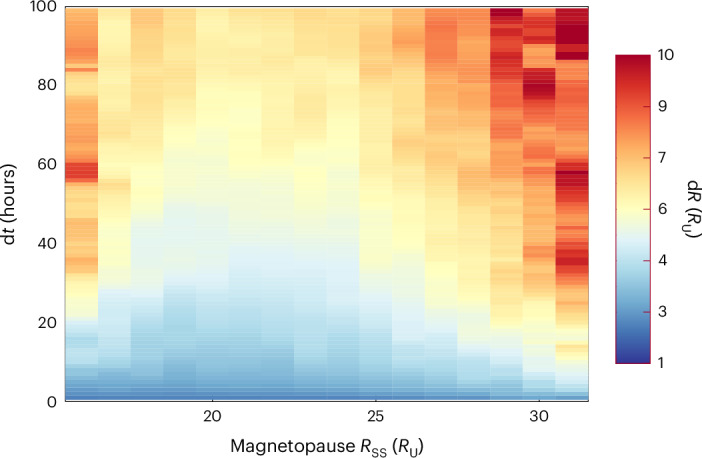
Time variability of the Uranian magnetopause. Estimate of how the magnetopause can vary in time and size (d*R*/d*t*) for a particular magnetopause standoff distance *R*_SS_. See  Methods for more details. Source data

**Extended Data Fig. 1:**
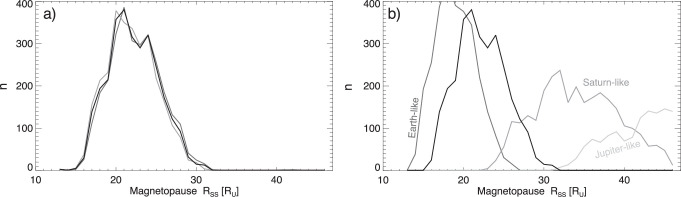
Variation of the subsolar magnetopause standoff distance estimation. Same as Fig. 3 in the main text but examples of how different α values used in Eq. 2 would vary the distribution. On the left in panel **a**) gray lines show α values of 5.68 and 5.72, while on the right, panel **b**) shows how α values of 6, 5 and 4.5 represent Earth-like, Saturn-like and Jupiter-like cases, respectively; compared to the fitted Uranus distribution of α equal to 5.7.

Based on the observed range of solar wind conditions, we present the expected probability distribution of magnetopause locations at Uranus in Fig. 3 (see Methods and Extended Data Fig. 1 for more details). The average magnetopause location is shown by the black dashed line, whereas the red dashed line shows the location at which Voyager 2 observed the magnetopause. There exists a very large range of expected magnetopause locations at Uranus. Furthermore, from this distribution we can estimate that a Voyager 2-observed magnetopause *R*_SS_ of 17.3 *R*_U_ or lower is expected to occur only 4% of the time at Uranus. This makes the results from the Voyager 2 flyby far from representative of average magnetospheric conditions at Uranus. Furthermore, the act of consistently compressing the magnetosphere for the days leading up to the flyby would drive internal dynamics that would then change the state of Uranus’s magnetosphere, making it further unrepresentative of what would be expected under average upstream conditions. Based on Fig. 4 (see Methods for more details), even within the timescale of the Voyager 2 flyby at Uranus through the planet’s inner magnetosphere (~1–2 days), *R*_SS_ may have changed by as much as 5–6 *R*_U_. This variability might influence the magnetic field mapping, but this is unlikely to change the conclusions of Connerney et al.^12^.

Furthermore, we analysed other CIRs that are evident in Fig. 2, to try to understand how variable *P*_dyn_ may have been while Voyager 2 was inside Uranus’s magnetosphere (Supplementary Information and Supplementary Figs. 3 and 4). The CIR profiles show that there is likely to have been some variability in *P*_dyn_ which may have temporarily compressed or expanded the Uranian magnetosphere during the flyby. Therefore, the suggestion that the magnetosphere may have moderately expanded during Voyager 2’s closest approach at Uranus^11^ is possible. However, Uranus’s magnetosphere must have been further compressed while Voyager 2 was inside the magnetosphere because *P*_dyn_ is higher upon exiting the magnetosphere than upon entering. Therefore, the magnetosphere would have been in a state of compression in comparison with the conditions a few days before the flyby started.

## Effects on the Uranian magnetosphere

Magnetospheric compressions can trigger a range of dynamic processes. For example, such compressions have been shown to increase Saturn’s kilometric radiation and Earth’s auroral kilometric radiation (AKR) intensity and extension to lower frequencies^13–16^. The same has been reported for Uranus’s kilometric radiation from the Voyager 2 flyby^9^, which shows a similarity between all three planets. During one solar wind compression event studied at Saturn where the kilometric radiation intensity increased, evidence for magnetotail dynamics, including magnetic reconnection was reported, featuring plasma injection from the tail to the middle magnetosphere as well as reconfiguration of the magnetic field^17^. Magnetospheric ‘quiet’ conditions have also been found to occur during extended solar wind rarefaction regions at Saturn^18^. Effects of compression at Saturn include the solar wind dynamic pressure initiating a global response from the aurora^19,20^ and magnetic reconnection at the dayside magnetopause^21^ driving magnetosheath plasma into the magnetospheric cusps^22,23^. Compression events at Saturn have been shown to result in sustained magnetotail reconnection, driving hot plasma towards the planet^24^. Magnetohydrodynamic simulations at Saturn show that plasmoid formation and release increases in frequency with increasing *P*_dyn_(ref. ^25^). At Earth, AKR has been shown to occur during pressure enhancements^26^ and it is also correlated to substorm activity^27^. In addition, substorms at Earth cause large variability of the radiation belts^28^. Investigations of magnetotail conditions at Uranus found evidence of magnetic substorm activity similar to Earth's^29,30^, most likely caused by a compression of the solar wind. Considering some of the similarities between the magnetospheres of Earth, Saturn and Uranus, it is therefore important to consider the series of possible effects that this sustained period of increasing solar wind dynamic pressure would have had on Uranus’s magnetosphere during the Voyager 2 flyby.

Estimations of mass loss from plasmoids^31^ have suggested very low values of just ~0.007 ± 0.004 kg s^−1^, which is 30–50% of an atmospheric plasma source of ~0.02 kg s^−1^ (estimated by Bagenal^32^). If there had been a series of plasmoids released regularly just before Voyager entered the magnetosphere (triggered by the magnetospheric compression) it would possibly explain the apparent emptiness of the magnetosphere and would imply a substantially higher average mass loss—not necessarily precluding a source of mass loss from either the rings or moons.

Furthermore, at Saturn, Thomsen et al.^24^ found that, under prolonged intervals (that is, several days) of high solar wind dynamic pressure, the tail plasma sheet is eroded away and the plasma composition is altered. The internally generated water-group ions (from the icy moon Enceladus) are lost from the system, and the plasma becomes dominated by lighter ions instead. The lack of heavy ions at Uranus’s magnetosphere has been invoked for a lack of an internal plasma source and to argue that the Uranian moons are not active. Potentially, plasma loss at Uranus during this compression event may have contributed to the loss of heavy ions; it essentially may have emptied the magnetosphere of its plasma and earned Uranus the reputation as a ‘vacuum magnetosphere’^30,33,34^.

Compression-induced magnetotail activity for the week before the flyby may also have resulted in the Voyager 2 observation of Uranus’s unexpectedly intense electron radiation belts. The existence of such intense electron radiation belts^35^ was a major mystery given the very low magnetospheric plasma densities observed during the flyby^33,34^. As discussed, the atypical magnetospheric compression may have led to the lower than usual magnetospheric plasma densities observed by Voyager 2. However, it is also important to mention that, at Earth, geomagnetic activity can inject fresh particles (that is, a ‘seed’ population) from the magnetotail into the outer radiation belts, which are then energized to higher energies^28,36^. During periods of increased AKR (similar to the enhanced Uranus’s kilometric radiation that was observed by Voyager 2), an enhancement of ~1 keV electrons (that is, the seed population) are generally observed at Earth’s radiation belts^37^. Terrestrial substorms are also more likely to increase the radiation belts at Earth; these enhancements can last for days^28^.

At Earth it has been found that during the arrival of CIRs and subsequent magnetospheric compression, higher fluxes of relativistic electrons are produced compared with storms driven by the arrival of coronal mass ejections^38^. Similarly, at Saturn, CIRs are a major driver of electron radiation belt modulation with higher intensities and energies observed during their arrival^39,40^. Did Voyager 2 arrive towards the end of such an event and consequently observe an unusual and enhanced electron radiation belt?

The solar wind conditions at Uranus are also important for theoretical characterization of dayside magnetopause reconnection driving the coupling between the solar wind and the magnetosphere. Masters^4^ estimated that magnetic reconnection at Uranus is ‘severely suppressed’ when compared with Earth’s magnetosphere, owing to the properties of the solar wind in the outer heliosphere^41–43^. However, such a conclusion was based on the high solar wind *P*_dyn_ conditions observed by Voyager 2 just before bow shock crossing, which we have just shown are not representative of the average conditions expected at Uranus. Our result, therefore, agrees with the findings of Gershman and Dibraccio^44^, who suggested that reconnection in the Uranus system is more favourable than previously predicted.

Figure 2 also reveals that variability in the solar wind is modulated on a timescale of approximately one solar rotation period, with corotating interaction regions (higher *P*_dyn_; disturbed solar wind) recurring approximately every 27 days. This means that, at least during solar minimum, Uranus regularly goes through alternating periods of very high and low upstream *P*_dyn._ This may reasonably be expected to have important consequences on magnetospheric dynamics. Voigt et al.^11^ originally suggested that the Uranian magnetosphere may go through cycles of being ‘open’ and ‘closed’ to the solar wind. Cao and Paty^6^ observed such cycles in their magnetohydrodynamic simulations of Uranus’s magnetosphere, and suggested that the magnetosphere is ‘switch-like’, where reconnection ‘switches’ on, opening the magnetosphere in cycles. Jasinski et al.^45^ proposed that such ‘open–closed’ or ‘switch-like’ cycles are due to the large dipole tilt when Uranus (or Neptune) enters the phase of a pole-on facing magnetosphere, which suggests that reconnection will always occur during this phase due to the antiparallel magnetic configuration near the subsolar magnetopause.

From the results in Fig. 2, we suggest that the Uranian magnetosphere may well have had two cycles at the time of the Voyager 2 flyby: the first varying on a diurnal timescale, due to the ‘switch-like’ or ‘open–closed’ processes mentioned above, and the second due to the varying solar wind conditions that change quasiperiodically on timescales of a solar rotation. The magnetosphere is expected to vary periodically between states of being expanded and compressed, and many of the magnetospheric phenomena related to compression and expansion would therefore also be expected to vary on solar rotation timescales.

Finally, we emphasize that the main purpose of our analysis is to demonstrate how drastically different the results from Voyager 2 could have been at Uranus, and how we should be cautious about the conclusions drawn from the Voyager 2 flyby; it remains to be seen how strong an effect magnetopause compressions have in driving Uranus’s magnetosphere. However, we also note that, if Uranus usually has a more plasma populated magnetosphere (that is, not a ‘vacuum’ magnetosphere), then magnetopause currents will become increasingly more important, which will act to expand the magnetosphere. This means that our average magnetopause location of ~22 *R*_U_ and a maximum location of ~34 *R*_U_ (Fig. 3) might be conservative lower estimates.

We also highlight the importance for modelling efforts (such as magnetohydrodynamic simulations of Uranus’s magnetosphere^5,6,46^) of using upstream conditions that are more representative of the solar wind, to better understand this planet’s space environment.

## Detection of subsurface oceans

A major outstanding question at Uranus is whether the major Uranian moons are present-day ocean worlds. The inner three of the five major moons of Uranus (Miranda, Ariel and Umbriel, located at 5.1, 7.5 and 10.4 *R*_U_ from Uranus, respectively) all orbit well within the magnetosphere, and therefore would exhibit a substantial magnetic induction response if electrically conductive oceans are present beneath their surfaces^47,48^. The two outer moons, Titania and Oberon (located at 17 and 22.8 *R*_U_ from Uranus, respectively), are more likely candidates for harbouring liquid water oceans^49^; however, detecting these oceans through magnetic induction would be difficult owing to the weak strength of Uranus’s magnetic field at their orbital distances. These moons are thought to orbit very near to or out of the magnetopause boundary, which can disrupt the predictable periodic nature of Uranus’s rotating planetary field in the frame of the moon and thus complicates magnetic induction investigations. This has been suggested to be a potential problem with future mission trajectory planning^7^, because it will be highly challenging to measure any magnetic induction signals when the moons are located in the magnetosheath.

**Fig. 5:**
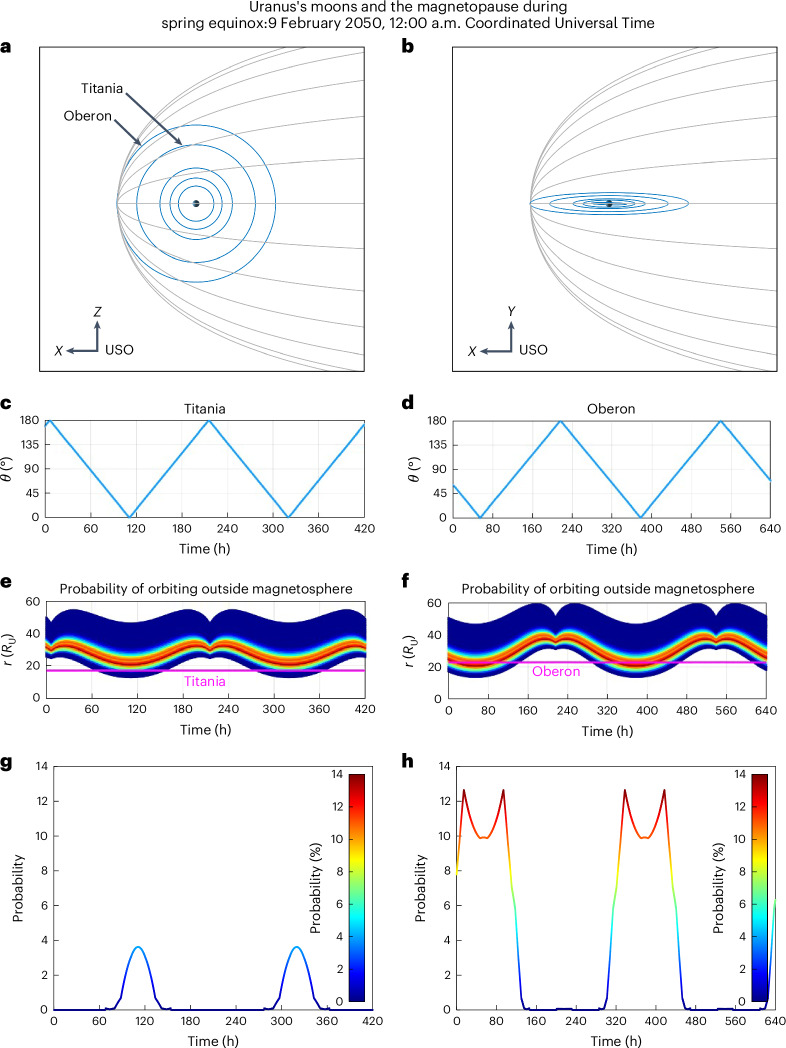
The moons Oberon and Titania and their location with respect to the Uranian magnetopause. Expected positions of the outer major moons Oberon and Titania with respect to the magnetopause for a future flagship mission expected to arrive near equinox in the late 2040s and early 2050s. **a**,**b**, The Uranian major moon system (orbital locations in blue) with respect to the Uranian magnetopause (grey lines) shown in the X-Z plane (**a**) and the X-Y plane (**b**) (see Methods for more details). **c**–**h**, Analysis for the moons Titania (**c**,**e**,**g**) and Oberon (**d**,**f**,**h**). **c**,**d**, The orbital angle between the moon and the Uranus–Sun line with time (that is, phase). **e**,**f**, The orbital radial distance of the moon (magenta), and the expected probability of the closest magnetopause location shown as a colour scale. **g**,**h**, The probability of the moon being outside the magnetopause as a timeseries for a window of time spanning two orbital periods of the moons. The colour scale shown in **g**,**h** is also the same colour scale for the probability distribution of the magnetopause shown in **e**,**f**. Source data

From our analysis (detailed in Methods and shown in Fig. 5 below), we conclude that magnetic sounding of these moons should not place any considerable constraint on spacecraft orbital trajectories because Titania and Oberon are expected to have relatively low likelihoods (<4% and <13%, respectively) of exiting the magnetosphere (and only for a small segment of their orbit when they are closest to the magnetopause). This result is also important for understanding the plasma interaction at these moons. A magnetosheath or solar wind plasma interaction is likely to be very rare at these moons, with a moon–magnetosphere interaction being the dominant scenario for any coupling between the moons and the local plasma.

## Conclusions

We have demonstrated that Voyager 2 observed the Uranian magnetosphere during a highly atypical and compressed state, with a subsolar standoff location of ~17 *R*_U_. Such a close magnetopause location is expected only <5% of the time, with an average magnetopause expected at 22.2 *R*_U_. Had Voyager 2 arrived at Uranus a week earlier, the magnetopause would have been crossed at ~28 *R*_U_ owing to the much weaker solar wind dynamic pressure at that time. Subsequently, a drastically different magnetosphere would have been observed. The magnetosphere of Uranus was continuously compressed (from ~28 to ~17 *R*_U_) for the days leading up to the Voyager 2 flyby, which is likely to have affected the magnetospheric dynamics and the resulting Voyager 2 observations obtained within the magnetosphere. This may have affected the magnetospheric plasma density and composition, and the radiation belts, as well as magnetotail dynamics. This would explain the presence of an unusually (and, up until now, inexplicably) intense electron radiation belt in addition to Uranus’s ‘vacuum magnetosphere’; both of which are likely to be transient features of Uranus’s magnetosphere. Because of the unrepresentative nature of the solar wind conditions encountered during the Voyager 2 flyby, the observed magnetospheric conditions were likely to be unrepresentative of the average state of the Uranian magnetosphere.

Owing to the variation of the solar wind at Uranus, we suggest that there may be two magnetospheric cycles during solar minimum. The first varying on a diurnal timescale (~17 hours) due to the extreme dipole tilt and obliquity. This cycle will exist regardless of solar cycle. The second magnetospheric cycle will vary on timescales of a solar rotation (~27 days) due to the quasiperiodically varying solar wind conditions during solar minimum. Understanding the variability of Uranus’s magnetopause is also important for future mission planning. Our estimates show that there is a very low chance that Titania and Oberon (the outermost major Uranian moons) orbit outside the magnetopause. This is important for magnetic induction studies that will attempt to determine whether subsurface oceans are present at the Uranian moons, as well showing that a magnetosheath or solar wind plasma interaction at these moons is expected to be rare.

We highlight that our understanding of the Uranus system is highly limited, and our analysis shows that any conclusions made from the Voyager 2 flyby are similarly tentative. We suggest that discoveries made by the Voyager 2 flyby should not be assigned any typicality regarding Uranus’s magnetosphere.

## Methods

### Magnetopause subsolar standoff (*R*_ss_)

Voyager 2 crossed the magnetopause at approximately 10:07 ut on 24 January 1986 (ref. ^50^). Using the publicly available SPICE toolkit^51^, this corresponds to a location of *X*_USO_ = 15.783994, *Y*_USO_ = 8.684153, *Z*_USO_ = −0.632197, *R*_U_; where USO is the Uranian-solar-orbital coordinate system, in which *X* points towards the Sun, *Y* points in the direction opposite to the orbital velocity vector (that is, points duskward in a traditional magnetosphere) and *Z* is positive in the northward ecliptic plane. This means that Voyager 2 passed at an angle of *θ* ≈ 29° away from the subsolar magnetopause (an angle of 0° would mean a subsolar magnetopause crossing). Ness et al.^50^ reported a subsolar standoff distance of 17.8 *R*_U_, Bridge et al.^1^ reported a standoff distance of 18.04 *R*_U_ and Voigt et al.^11^ reported the subsolar standoff distance to be 18 *R*_U_.

To estimate how the magnetopause distance *R* from the planet varies with angle *θ* away from the subsolar point, we used the simple Shue et al.^52^ functional form which has been used extensively at all the planets with intrinsic magnetospheres^52–56^.

1 $$R={R}_{{\rm{SS}}}{\left(\frac{2}{1+\cos \theta }\right)}^{\xi }$$

where *ξ* denotes the dimensionless flaring parameter of the magnetopause, with an *ξ* closer to 1 having a larger magnetopause distance at the flanks than a *ξ* value closer to 0 (where the flank magnetopause would flare towards the planet). The value reported^1^ of *R*_SS_ = 17.8 *R*_U_ would require an *ξ* = 0.2, which is not realistic. Instead, we used values reported from magnetohydrodynamic simulations of the Uranian magnetopause by Cao and Paty^46^. Due to Uranus’s large obliquity and dipole tilt, the magnetopause will not be as symmetrical in the noon–midnight meridian as other planetary magnetospheres, and so these authors investigated how the magnetopause at Uranus varies with rotation. Their results show that the flaring parameter *ξ* will vary between 0.45 and 0.75. We used a middle value of 0.6 for our calculation, which produced an *R*_SS_ = 17.3 *R*_U_ (for a *P*_dyn_ of 0.0184 nPa). We note that a flaring parameter range of 0.45–0.75 produces an *R*_SS_ range of 17.17–17.51 *R*_U._ This wide range, which is mostly closer to 17 *R*_U_ is why, for this article, we denote the *R*_SS_ during Voyager 2 as simply being close to ~17 *R*_U_.

The magnetopause subsolar standoff distance (*R*_SS_) can be approximated from the upstream solar wind *P*_dyn_ using a simple pressure-balance relationship, which has been validated at Earth, Jupiter and Saturn^52–58^.

2 $${R}_{{\rm{SS}}}=\sqrt[{\scriptstyle{\alpha}\atop}]{\frac{{B}_{0}^{2}}{2{\mu }_{0}{P}_{{\rm{dyn}}}}}$$

where *B*_0_ is the equatorial ‘surface’ magnetic field strength of the planet at the 1 bar level (23,000 nT (ref. ^1^)) and *μ*_0_ is the permeability of free space. We estimated *α* (a dimensionless compressibility parameter, which will vary depending on the internal thermal plasma pressure) using the Voyager 2 flyby values for *R*_SS_ and *P*_dyn_, which resulted in *α* = 5.7. Using this relationship, we could estimate what the magnetopause location was for the weeks leading to the Voyager 2 flyby. Propagation of the solar wind from the location of Voyager 2 to Uranus is not necessary here since the solar wind is not expected to vary notably within the 4–5 hours it takes for it to arrive at the planet. For an understanding of how the solar wind varies throughout the heliosphere (on interplanetary scales) we refer the reader to Masters^43^ or Gershman and DiBraccio^44^. Unfortunately, no other plasma measurements of solar wind at this radial distance exist, because Voyager 1 and New Horizons, which crossed Uranus’s orbital path, both had their plasma spectrometers switched off during this time.

Propagation of the solar wind *P*_dyn_ conditions across ~2 au (18.28 to 20.09 au) of solar wind data (that is, Fig. 2) is not required as this is the region in heliocentric radial distance within which Uranus orbits and therefore is local to Uranus. However, if we were to consider propagating (that is, normalizing) the solar wind data in Fig. 2 specifically to the heliocentric radial distance at which Voyager 2 completed its flyby of Uranus (that is, 19.1 au), then this would not change our results and conclusions. At most, when Voyager 2 is at the edges of this range in radial distances (that is, at 18.28 or 20.09 au), the solar wind *P*_dyn_ would change by 9% before travelling 1 au to reach Uranus, and this change would decrease as Voyager 2 is closer to Uranus. Please refer to the Supplementary Information for a more in-depth discussion as well as figures showing this in more detail.

The range of possible *R*_SS_ values for the Voyager 2 magnetopause crossing (from a varying *ξ* in equation (1) discussed above) will affect the possible ranges of estimated *R*_SS_ values that we calculate from solar wind *P*_dyn_ values (that is, Figs. 1d, 2d and 3). The variation in *R*_SS_ that the flaring parameter *ξ* introduces will correspond to a range of *α* values for the correlation of *R*_SS_ ≈ *P*_dyn_^−1/*α*^. In our analysis, we used a value of *α* = 5.7, but the range would be 5.68–5.72. We plot this in Extended Data Fig. 1a (left), which shows in grey how Fig. 3 would vary for different *α* values in this range. This fitting does not produce a substantial change in our results. For comparison, we also show how the magnetopause distribution would vary if we assumed an Earth-like (*α* = 6), Saturn-like (*α* = 5) and a Jupiter-like (*α* = 4.5) magnetopause in Extended Data Fig. 1b (right). An Earth-like estimation would give a magnetopause of 15 *R*_U_ for Voyager 2-observed conditions which is contrary to observations, whereas the Saturn-like and Jupiter-like examples are similarly unable to reproduce the observations.

The determination of *α* of the above relationship is an important result in itself, and it provides key new insights for the system. Both the magnetic and thermal pressures are important in determining the magnetopause location. At Earth, the magnetic pressure dominates and the subsolar point can be predicted through magnetic pressure alone. At Jupiter, the hot internally generated plasma pressure is as important as the magnetic pressure inside the magnetopause. This causes the Jovian magnetosphere to be distended during low *P*_dyn_ conditions. Although Earth’s magnetopause is more rigid in its response to changes in *P*_dyn_, Jupiter’s magnetopause is much more compressible and responsive to variations of the upstream *P*_dyn_. Saturn lies between Jupiter and Earth for magnetopause compressibility. Its centrifugal processes and internal plasma source (Enceladus) are weaker than Jupiter’s, which means that its magnetopause is less sensitive to variations in the upstream conditions than Jupiter’s but more so than Earth’s. Therefore, it is useful to make this analysis for Uranus, and curious to see that the estimates for *α* based on the Voyager 2 data fall somewhere between those for Saturn and Earth, which suggests that Uranus’s magnetopause response to solar wind dynamic pressure variations is an ‘in-between’ of Saturn and Earth, and much closer to being Earth-like. Whether this is an indicator of a possible (minor) internal plasma source (or lack thereof) is beyond the scope of this paper, but does leave a lot of questions open. We highlight that this fitting is based on a single crossing of the magnetopause, and that many more boundary crossings are required by a future orbiter to answer this question and to characterize the magnetopause accurately.

We also note that the diurnal variation of the magnetic dipole with respect to the solar wind flow (that is, the solar wind attack angle; SWAA) will affect the magnetopause standoff distance. Fortunately, the Voyager 2 flyby occurred during summer solstice conditions, which represents the most Earth-like configuration of the magnetosphere and the least varying solar wind attack angle at Uranus (51°–66°). In fact, Earth’s magnetosphere has a larger variation in the SWAA (56°–77°) at the same orbital phase. Had the flyby occurred at a different phase, our method would require analysis of diurnal variations at Uranus because the SWAA can vary substantially (up to 120° compared with 15° during Voyager 2) which would have large effects on the magnetopause standoff distance.

### Magnetopause time variability (d*R*_SS_/d*t*)

We provide estimates of the variability expected at Uranus (during the Voyager 2 era) for any particular magnetopause standoff location. Using the data in Fig. 2d, we estimated the average magnetopause variation (d*R*_ss_) over different timescales (d*t*). This is shown in Fig. 4, with d*t* extending from 1 hour variations (cadence of Voyager 2 plasma subsystem (PLS) data, which is available on the Planetary Data System (PDS) website; see Data availability statement for a link to the PDS) to 100 hours (5.8 Uranian days). We can see that the extreme compressed or expanded magnetopause locations are the least stable and vary substantially (d*R*_SS_ ≈ 5 *R*_U_) on short timescales (d*t* ~tens of hours). In comparison, the magnetopause location (R_SS_ ≈ 23 *R*_U_) is the most stable, with variations of d*R*_SS_ ≈ 1 *R*_U_ occurring on the same timescale.

### Moon–magnetopause analysis

Using the distribution of expected magnetopause locations, we estimated the probability that the moons Titania and Oberon orbit outside the magnetopause (Fig. 5). We first minimized the squared distance between the location of the moons and the generalized Shue et al.^52^ magnetopause surface model at each instance in time along the orbits of Titania and Oberon. We used a nonlinear solver to extract the *θ* that minimizes this distance, which was then used to calculate the radial distance to this point on the Uranian magnetopause boundary, for the range of subsolar standoff distances shown by the distribution in Fig. 3. These plots effectively show the probability of moon–magnetopause crossings. This was calculated for the equinox case, in 2050, which is approximately the expected time that a Uranus spacecraft could realistically arrive at Uranus. An extensive set of calculations accounting for different seasonal phases is outside the scope of this work, but would be an important future toolkit for future spacecraft planning. We also note that our analysis does not take into account possible effects from Kelvin–Helmholtz instabilities at the flanks of the magnetopause that might be present at Uranus^43,59^. The full extent of Kelvin–Helmholtz instability growth is not well understood at Uranus, and understanding its effects is beyond the scope of this paper. Figure 5a,b illustrates the geometry of the Uranian moon system in USO coordinates during spring equinox, February 2050, a time which could potentially be a realistic time of arrival for a Uranus flagship mission. Figure 5c–f illustrates the location of these moons (${{\theta }},{{r}}$; where *r* is the distance of the moon from Uranus and *θ* is the angle the moon makes with respect to the Uranus–Sun line) and the Uranian radial distance to the point on the magnetopause surface that is nearest to the moon as a function of time for the distribution of magnetopause subsolar standoff distances illustrated in Fig. 3. The window of time for each plot coincides with twice the orbital period of the respective moons (*T*_Titania _= 209 h, *T*_Oberon_ = 322.9 h).

### Instrument measurement uncertainties

Table III in the Voyager 2 Plasma Spectrometer instrument paper^60^ (that is, the instrument which we use for our analysis), give values for the expected density error and velocity errors (which are required for *P*_dyn_) that are exceptionally small (error on velocity ~0.3%; error on density ~3%). This is because the solar wind Mach numbers are high in the outer solar system, which is beneficial for a Faraday cup instrument which makes up the plasma subsystem on board Voyager 2. Therefore, the measurements will not be within instrument errors.

## Supplementary information

Supplementary Information Supplementary Figs. 1–4 and Discussion.

## Source data

Source Data Figs. 1 and 2 Solar Wind measurements made by Voyager 2 while at Uranus’ orbital location, including the expected corresponding Uranian magnetopause subsolar standoff location.

Source Data Fig. 3 Uranus magnetopause location distribution.

Source Data Fig. 4 The variation of the magnetopause location (that is d*R*/d*t*).

Source Data Fig. 5 The expected probability that the moons Titania and Oberon orbit outside the magnetopause (with time).

## Data Availability

Voyager 2 data are available on the Planetary Data System (PDS: https://pds-ppi.igpp.ucla.edu/search/?t=Solar%20Wind&sc=Voyager_2&facet=SPACECRAFT_NAME&depth=1). The SPICE toolkit can be accessed at: https://naif.jpl.nasa.gov. Source data are provided with this paper.
