# Supplementary material for: The anomalous state of Uranus’s magnetosphere during the Voyager 2 flyby
Source: Nat Astron. 2024 Nov 11;9(1):66–74. doi: 10.1038/s41550-024-02389-3 (PMC11757144; doi:10.1038/s41550-024-02389-3)
Supplement: Supplementary file 1 — Supplementary Figs. 1–4 and Discussion. [file 41550_2024_2389_MOESM1_ESM.pdf]

nature astronomy

Supplementary information https://doi.org/10.1038/s41550-024-02389-3

The anomalous state of Uranus's magnetosphere during the Voyager 2 flyby

In the format provided by the authors and unedited

Supplementary Discussion

SI.1 Solar wind propagation (or normalization)

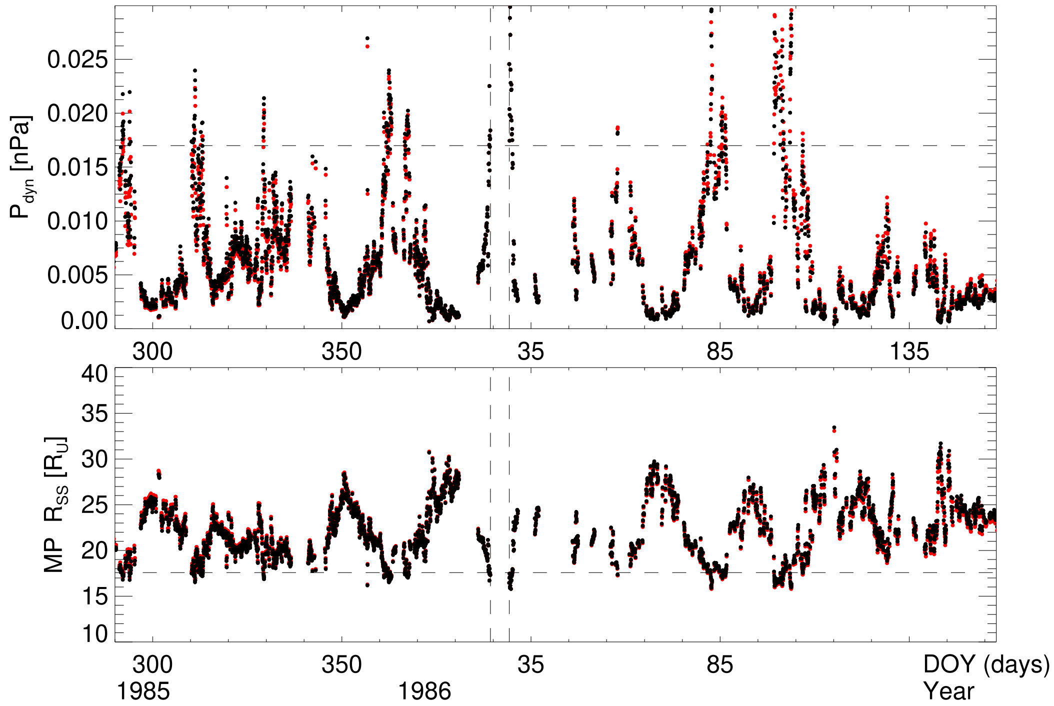

Supplementary Figure 1. This figure is in the same format as Figure 2 in the main text of the article however here we show possible propagation of the solar wind dynamic pressure and its effect on the magnetopause standoff distance, in red (original Voyager 2 data in black).

We have propagated the solar wind density values using a simple $1/r^2$ relationship to the location in heliocentric distance that Voyager 2 flyby completed at Uranus (i.e. normalized the solar wind data to 19.1 au, considering a possible change in the solar wind conditions due its expansion throughout the solar system), shown in Supplementary Figure 1 above. This propagation does not change the values significantly and does not change our conclusions. Furthermore, it does not change the magnetopause distribution shown in Figure 3 of the main text. Supplementary Figure 2 below shows how insignificant the magnetopause distribution would vary (grey) if we propagated the data.

Copyright 2024. All rights reserved.

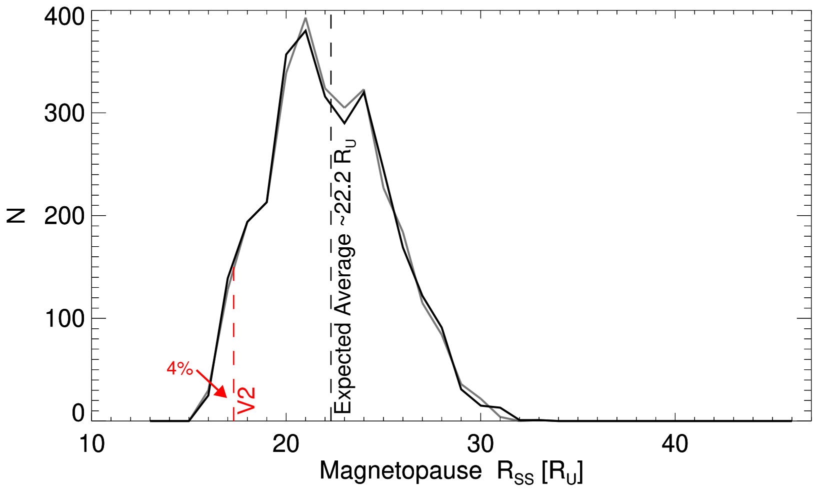

Supplementary Figure 2. This figure is in the same format as Figure 3 in the main text of the article however here we show the possible propagation of the solar wind dynamic pressure and its effect on the magnetopause standoff distance, in gray (original Voyager 2 data in black).

SI.2 Corotating interaction regions and their variations

We have attempted to deduce how much the upstream solar wind dynamic pressure (P_{dyn}) may have varied while Voyager 2 was inside Uranus' magnetosphere, by looking at other CIRs within the dataset of Figure 2 in the main text. The CIR that impacted Uranus during the Voyager 2 flyby is shown in the top panel in yellow, and four other CIRs are also shown below in Supplementary Figure 3. The CIRs are extremely variable and have different durations varying from 4 to 10 days. The flyby CIR lasted approximately 7 days. This CIRs show that there was most likely some variability in P_{dyn} during the Voyager 2 flyby, which may have compressed or expanded the Uranian magnetosphere moderately during the flyby (see Supplementary Figure 4). Therefore, the suggestion made by Voigt et al., 1987, that the magnetosphere may have expanded at Voyager 2's closest approach at Uranus, is possible.

However, we certainly know that Uranus' magnetosphere must have been further compressed during the Voyager 2 flyby since P_{dyn} is higher upon exiting the magnetosphere than upon entrance. Therefore, the magnetosphere would have been in a state of compression in comparison to the conditions a few days before the flyby started.

Copyright 2024. All rights reserved.

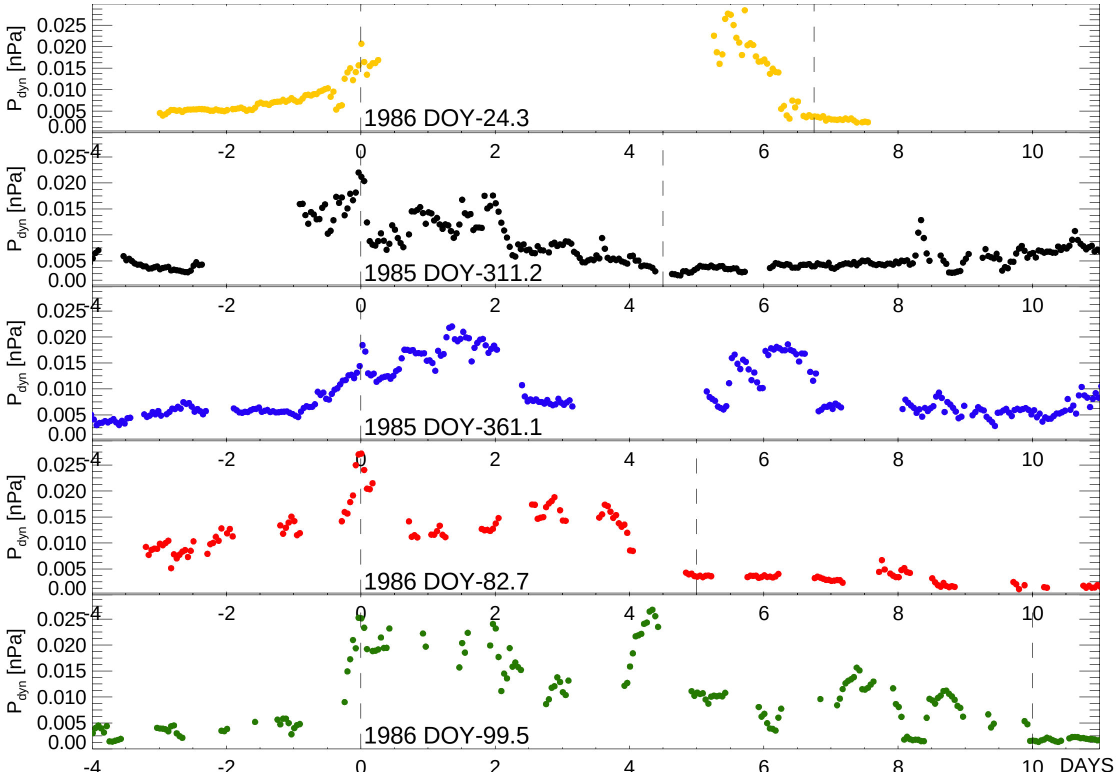

Supplementary Figure 3. This figure shows the corotating interaction region (CIR) that impacted Uranus as Voyager 2 was approaching Uranus, as well as four other CIRs. Time starts at the first dashed line (t=0) from the first peak in the dynamic pressure and is labelled accordingly. The second dashed line shows possible "end" times of each CIR.

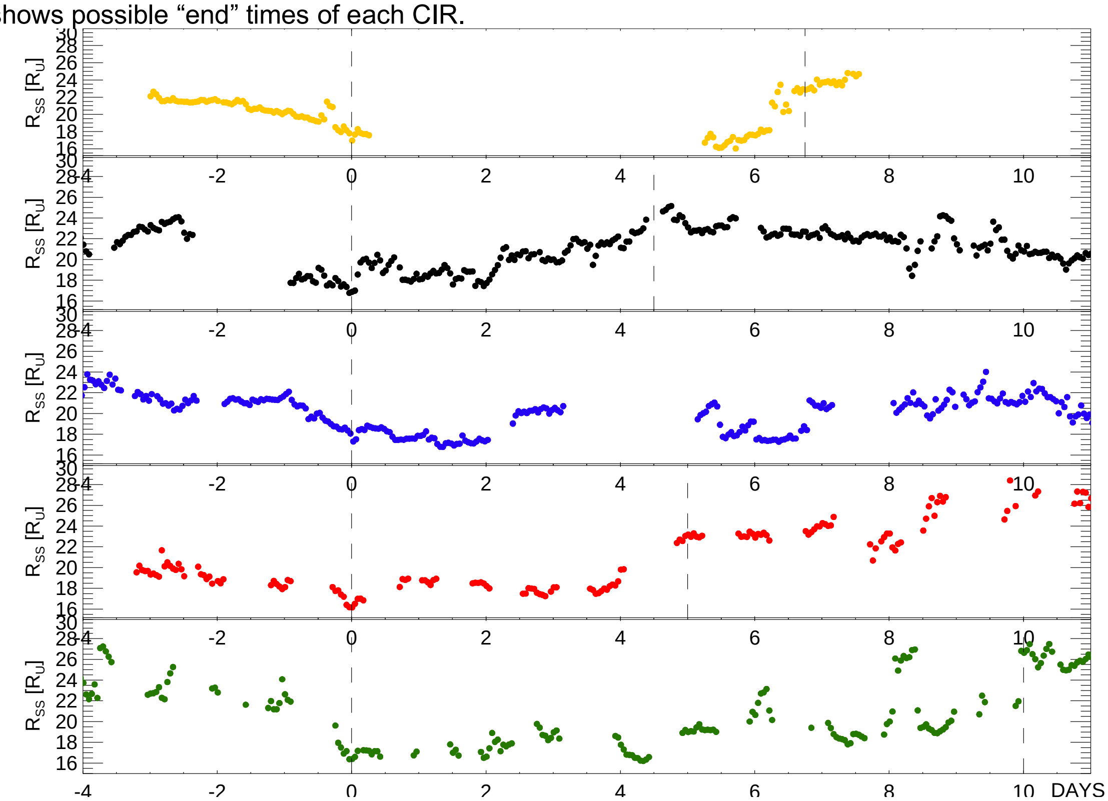

Supplementary Figure 4. The same as Figure SI-3 but showing the magnetopause subsolar standoff distance.

Copyright 2024. All rights reserved.

The research was carried out at the Jet Propulsion Laboratory, California Institute of Technology, under a contract with the National Aeronautics and Space Administration (80NM0018D0004).

References

Voigt, G.-H., Behannon, K. W., and Ness, N. F. (1987), Magnetic field and current structures in the magnetosphere of Uranus, J. Geophys. Res., 92(A13), 15337–15346, doi:10.1029/JA092iA13p15337.

Copyright 2024. All rights reserved.
